# Some Observations on the Climate of the West1Read at the 35th annual meeting of the Medical Association of Central New York, held at Syracuse, N. Y., October 21, 1902.

**Published:** 1902-12

**Authors:** John L. Heffron

**Affiliations:** Syracuse, N. Y., 528 South Salina Street.


### Some Observations on the Climate of the West.[1]

By JOHN L. HEFFRON, A. M., M. D., Syracuse, N. Y.

SOME one has said that "the pleasures of life consist of climate and the affections." This is more nearly true than most generalisations. Indeed, the resultant of these two factors, climate, or man's place in nature, and the affections, or man's place in society, is greater than is thus indicated. It would be easy to demonstrate that not only is man's pleasure in living, but his individual characteristics, his communal traits, his moral and mental status and his physical well being are largely influenced by climate and the affections. As physicians and climato-therapists we are chiefly interested in those forces that aid in the establishment and preservation of health. But in our studies of climate if we leave out the social features, the "affections," we make an error that will tend to diminish our usefulness to those who consult us. You remember the story of the old sea captain who at a "revival" meeting in a Methodist chapel failed to rise when all were asked to stand up who wanted to go to Heaven—the Heaven the preacher had eloquently portrayed in his sermon? A good sister approached him and said, "Why, Captain, don't you want to go to Heaven?" His reply was "No! why should I want to go to Heaven and sit naked on a cloud twanging a harp? I'm no singer and I wants my mates!"

Perhaps no better opportunity of studying the climates of some portions of the West and the social features of the various

---

1. Read at the 35th annual meeting of the Medical Association of Central New York, held at Syracuse, N. Y., October 21, 1902.

resorts was ever presented than was given to the members of the American Climatological Society in their trip this summer to the Pacific coast. It was my good fortune to be a member of this party and it gave me an opportunity to correct some false impressions and make some observations which may possibly interest those who employ climate as a therapeutic measure. The itinerary was arranged conjointly by the president, a Denver physician, the secretary, a Philadelphia climatologist, some California friends, and the Raymond & Whitcomb Co. and was made to include the greatest possible points of peculiar interest in New Mexico, Arisona, California, Utah and Colorado.

Right here it might be asked with propriety, "What went ye out for to see?" What do we mean by climate and climatic resorts? I presume Webber's definition, "that combination of atmospheric conditions and of the earth's surface determining the suitability of a region for the life and health of animals and plants" is as good as any. But it may be said with truth that all regions of the earth's surface, or nearly all, are such as to make the conditions of merely living possible to both animals and plants of many species, if not for all. From a therapeutic standpoint, therefore, we must give a more limited definition to climate. A therapeutic climate is such a combination of atmospheric conditions of the earth's surface as makes healthful living most highly possible for those children of men who have the least physical resistance either innate or acquired. In the Walker-Gordon laboratories, milk, the natural food of infants, is separated into its elements and recombined in such a variety of ways as to meet the demands of any vagary of digestion an infant may manifest. If we could compose the various elements of climate, temperature, light, moisture, altitude, topography, soil condition, and the like, in such a way as to meet exactly the needs of various patients it would be a consummation devoutly to be wished, but, working under natural conditions, as we must, we can but search the earth's surface for those regions in which are combined the greatest number of desirable elements with the least of the deleterious factors for each condition demanding climatic changes. The climatic conditions observed can perhaps be best illustrated by reviewing seriatim the different places visited and noting their natural advantages.

At Dodge City, Kansas, we woke up one morning to find our train stalled and unable to proceed because of a flood which was coming down the Arkansas River and which had swept away bridges and miles of track on the route we were to take into New Mexico. This interruption caused a delay of forty hours, and while it necessitated a change in our program it was not without interest. The Arkansas River at Dodge City under normal conditions in June was a shallow stream in which the omnipresent bare-footed boys disported themselves without getting more than their feet wet while they waited for the flood. The assurance of the train conductor, who had run ahead of the flood, that a cloud burst had occurred in the mountains, and that a wall of water fifteen feet high was surging down the gorge and that at Dodge City the rise would be at least five feet caused the ranchmen along the stream to hurry away their cattle and their families to high ground. This incident is not remarkable but the alternation of flood and drought as prevailing conditions is not observed in the East. Many so-called rivers were crossed in different states in which there was not a drop of water to be seen, only a sandy bed carefully spanned with heavy bridges. On asking why they called these reminders of the fact that water had sometime run there, rivers, the reply was, "Why, the water is there, but it is underground." And it is so. In any of these beds a pipe can be driven and water obtained at different depths, showing that they are natural drainage canals, though filled to the brim only during flood time. As this is one of the disadvantages generally seen, and one which the recent legislation providing for the storage of water is intended to overcome in a measure, it is worthy of mention. When one has seen what miracles can be produced in these desert regions by water and industry, one can but feel that at least as much help should be extended by the general government in the regulation of the water supply of these arid regions as is given for the improvement of rivers and harbors in the more fortunate East.

This is only the first of the anomalies to an eastern observer —rivers that are invisible except in flood, but rivers just the same. Babbling brooks and running streams are a curiosity, and were seen by us only in lofty valleys where snow-capped peaks were near enough to suggest the source. This condition of things, the absence of surface rivers and large bodies of fresh water in lakes and ponds, of course has a great influence on the climate. The humidity of the atmosphere, as a rule, all through the west is very low, and this is one of the advantageous features of the climate. As a corollary, fogs and mists and heavy dews are never seen in inland places.

Our extra forty hours in Kansas, memorable for many reasons, shortened our stop in New Mexico. Visits all too brief were made at Las Vegas and at Albuquerque, but still they permitted some observations that were of value. It will be remembered that the medical officers of the United States government have selected New Mexico as the seat of two sanitoria for the treatment of soldiers and sailors suffering with respiratory diseases. The air was dry and pure, the sunshine flood the world, an altitude of from 5,000 to 7,000 feet gave light atmospheric pressure, the heat was comfortable summer heat and the soil was dry and porous. These conditions are generally prevalent and uniform. It is stated that the annual percentage of sunshine is over 73 per cent. and that the summers are a little cooler than those of New York, while the winters are a little warmer than at Charleston, S. C. At Las Vegas with an elevation of 6,384 feet the average noon temperature in winter was 40°, in spring 55°, in summer 80° and in the fall 60°. A pure, dry, rare atmosphere is desirable in many cases of lung trouble. But there are some cautions that should always be given patients sent to such a climate. I am aware that much is said of the benefits to be derived by those suffering from heart disease of moderate severity by such climatic conditions. I am not ready to dispute them, but at Albuquerque I saw two young men whom I had seen here in consultation and whose lungs were in such an advanced state of disease that I advised against sending them to a resort so remote from their friends. Both of them had sound hearts in Syracuse. One of them had been so ill that little exercise had been possible, the other had in two years time greatly improved in his pulmonary condition and in his general health, and had been imbued with the idea that he should take as much physical exercise as he could endure. He told me that the day before he had ridden 100 miles on horseback. On examination I found a greatly enlarged heart with a loud mitral systolic murmur.

A prominent attorney who was sent to Colorado from New York in his young manhood for lung trouble, and who had regained his health and had become very prosperous happened to come under my care in an eastern visit during which he had broncho-pneumonia. For several years he paid me annual visits, and at each I urged his removal from the altitude at which he lived because of the strain upon his heart, which I considered gave evidence of myocardial degeneration. His interests were so tied up with his western home that this seemed impossible to him. He died suddenly while ascending the steps to his office. Of course, both of these cases may be considered as results of other causes than a rarified atmosphere, and I do so consider them, but I think there will be little question but that the conditions are more apt to be developed at an altitude of a mile above sea level than at the sea level, all other conditions being the same. The wise physician, then, will warn his tubercular patient against severe physical exercise in a high altitude and he should do more. He should refer his patient to some physician in the place to which he is sent whom he knows to be skilful and of sound judgment.

If you imagine that the physicians in the west prescribe only air and sunlight you are sadly mistaken. While in every center there are a few physicians who are intelligent and educated and conservative, there are in every climatic resort at the same time a host of quacks that can put to blush any of this species practising in the east. I consider placing the patient under an intelligent and reliable physician immediately upon his arrival in the city of his destination an absolute necessity. It is at first, before he has learned the climatic peculiarities, that he most needs guidance and careful observation. You would hardly credit the instances of the grossest and most unscrupulous impositions on the sick which I could relate to you. The greatest pity of it is that irregular and quixotic methods are not confined to those practising without the degree of some recognised school of medicine, but are indulged in by some who have a high local reputation. So that Polk's Directory is not a safe guide. The practice of sending patients away to a climatic resort with a medicine case and an order to keep you posted about the progress of the case is absolutely wrong. Such a patient is practically certain to get into the hands of a medical fakir, or to commit some costly error.

In Albuquerque we noticed that slight currents of air through the streets blew up considerable dust, and my young friends told me that regularly every afternoon the wind came down the valley in sufficient strength to make a dust storm that was very disagreeable while it lasted. Of course that is not desirable though we met one physician who very earnestly contended that it was beneficial because clean dust simply irritated the mucous membrane mechanically, and in its expulsion the lungs were purged of the discharge from the specific disease more perfectly than without such mechanical irritant. The dust storms in these regions are not entirely confined to cities. Vegetation is possible only along streams or where irrigation can be practised, and we had daily exhibitions of whirling sand on the open in our progress through the country. In fact, I think that our party would vote that the dust encountered in New Mexico, Arizona and inland California was about the only disagreeable thing we met. Of course we have dust, and possibly under similar conditions we would have as much, but my memory of its all-pervasiveness, its penetrating qualities and its sharp sting on mucous membranes classifies it as a different article. These disagreeable accompaniments of an otherwise ideal climate can be minimised, if not entirely eliminated, by care in the selection of towns so situated as to be free from currents that can be the bearers of dust, and New Mexico, like most of the western states, has such a variety of topographical features that all of the climatic elements save moisture can be varied by change in location.

In Arizona our stops were at the Grand Cañon of the Colorado, Prescott and Phenix. This gave opportunity of observing conditions in northern and southern parts of this territory. The elements which are constant throughout Arizona are dry air and a maximum of sunlight throughout the entire year. The temperature in northern Arizona is lower than in the southern parts of the state due to a greater elevation and the greater nearness of snow-capped mountain ranges. But in Phenix, when in the June day of our visit the thermometer registered 100°F. at the government observation station, the effect of the heat is far different than it is with us. While the temperature on that day was 100° F. the humidity was but 2 per cent. and the result was, that after a drive in and about Phenix of several hours in the middle of the day, and an elaborate dinner at 2 p.m. none of us were in the least degree unfavorably affected by the heat, and could not realise from our sensations that the thermometer had registered so high. It was here that we first observed the marked difference in sensible heat between the sunlit places and the shade. It is a difference so marked that no eastern man can properly appreciate it without a personal experience. Any object that casts a shadow is a cool retreat, and it realised to us the meaning of that oft-quoted passage "the shadow of a rock in a weary land." The same conditions were observed throughout the trip. It was curious to see the birds make use of this discovery and seek for their refreshment the shadow of anything, even a telegraph pole. The same causes which produce this marked difference are productive of the marked difference between the temperature by day and by night. Cool nights are the rule, and this difference is still more marked in those regions where there is an alternation of currents from the mountains and from the plains or from the ocean.

The hospitalities that were showered upon us in Arizona were typical of what we received wherever we went. Nothing that the residents of any of the places visited could contribute to increase our comfort and enable us to appreciate to the full the advantages of their home cities was left undone, so that it can be said with certainty that no stranger will be neglected in any of our western climatic resorts. There is a community of interest and a general friendliness which is not purely commercial and it prevails in all ranks of society. If there is an exclusive set we were not allowed to know it, and if there had been, some of the party were distinguished enough to have appealed to it and to have been marked by the lion hunter in society. No such thing was observed. The literature of Arizona climate is full and that compiled by the government stations is accurate and reliable. The territory offers a wide selection of most admirable resorts for both summer and winter and is without question one of the most favorable places for those afflicted with pulmonary diseases.

In the enormous state of California our stay was much longer and our stops more numerous. Of the inland cities, or foot-hill towns, Redlands and Riverside were types and cities more rich in natural advantages and in the beauty of landscapes, teeming with fruits and flowers, man has rarely seen. At Loma Linda, near Redlands, a modern sanitarium for the care of nontubercular patients has been provided that offers a delightful home with every provision for the modern and scientific treatment of the weak and weary and for the surgical care of those from nearby cities. In Strawberry Valley in the San Giorgione mountains, which readers of Romona will remember was selected by Alessandro as the last stand against the invasion of the white man, Idylwild, a modern sanitorium for the scientific treatment of incipient tuberculosis, has been located and admirably equipped by a company of physicians mostly residents of Los Angeles. Idylwild has an elevation of 5,500 feet and is situated in a romantic valley wooded with gigantic sugar pines and surrounded on all sides by the towering peaks of this spur of the coast range. Its situation and natural advantages are unsurpassed. Under the intelligent management which it enjoys it bids fair to be one of the notable institutions of its class. It can be said of California in general that the tuberculous patients are no longer solicited. And I think that the testimony of our party would be that except in such choice localities as has been just described the climatic conditions are not ideal for the cure of tuberculosis. On the other hand, after the lesions of phthisis have been healed, no more ideal spot for a permanent residence could be found than is offered by the many beautiful places in Southern California. Of the coast cities many were visited from San Diego in the south to San Francisco. The climate in general is very similar, varied only by the closer approximation of the seas on one hand or the mountains on the other. The temperature is equable but never high. The difference between the temperature of sun and shade and of day and night amounts to more than twenty degrees in many places. Those who imagine that Southern California is a region of eternal summer are doomed to disappointment. The summer weather is much like our late September, and fall clothing and an overcoat are indispensable for comfort. The nights are so cool that lawn parties and the enjoyment of piazzas in the evening are never thought of by those acclimated. To the neglect of precautions against these changes are traceable many of the acute illnesses from which tourists not infrequently suffer. The winters are hardly different from the summers, and it is to the great contrast between the inclement weather of the eastern seaboard and the almost summer weather of California that the ever-increasing popularity of this land of varied charms is due as a winter resort. The freedom from marked changes in weather, the low degree of humidity, the delightful scenery and the opportunity for outdoor life make California the ideal home for the nervous and those exhausted by the cares of business and of social life.

In Utah our only long stop was made at Salt Lake City. The peculiar beauties of this city are familiar to most of you, for no city has been more thoroughly written up than this. It has a dry, cool climate, free from frequent or severe storms, and its elevation of 4,350 feet, with its great amount of sunlight makes it an appropriate place for many diseased conditions, while the annual rain fall of 16 inches insures a fertility of its fields without so much attention to irrigation. The fact that it is a business center and surrounded by arable fields gives an advantage to many who in seeking a more desirable climate are compelled to earn their living from the start.

The rugged charms of Colorado were revealed to us by the inspiring trip on the Denver and Rio Grande railroad. At Glenwood Springs we found a most delightful climate. The celebrated waters and the hot springs, the bathing facilities and the fine hotel combine to make this place dear to memory. For those suffering from hepatic and rheumatic disorders the advantages here offered are hard to excel, while the lovers of noble mountain scenery will find a never failing source of delight.

Colorado has a greater reputation as a health resort for tuberculous patients than any other state in the Union, and we believe it is well deserved. The whole state has an altitude of from 4 to 10,000 feet while from the mountain ranges 130 peaks lift their sublime heads above an altitude of 13,500 feet. Because of this we have an atmosphere of low pressure. The air is dry and clear and bracing. Fogs and clouds are almost unknown and sunshine is abundant. The temperature varies from a mean of 30° in the winter to about 75° in the summer, but excesses in either direction are not unknown. However, the relative low humidity makes the sensible heat and cold much easier to bear. These conditions and the remarkable results obtained deservedly give Colorado a reputation unequaled for the outdoor treatment of tuberculosis. Colorado has the great advantage of the residence of a body of medical men of more than average intelligence, many of whom are themselves examples of the curative powers of the climate. The large cities are no longer the most desirable localities for patients, tho' Denver and Colorado Springs are so laid out as to be free from crowding and present ample air space about the individual homes, which are the types of residences, and are furnished with unusually good sanitary conveniences. For those infected with tuberculosis who have an equable nervous condition and who have inherited and maintained a good circulation and who have been born to such a succession of seasons as we in Central New York are accustomed to, I know of no more favorable locality for a cure than Colorado offers. On the other hand, I should hesitate to send there a tuberculous patient with a rapid, feeble pulse or one of unstable nervous endowment.

The various advantages of these health resorts have been described in such exaggerated language, and the stories of the miraculous cures effected have been so broadly advertised that many have gone thither in conditions which were absolutely hopeless and only to die earlier than they would have done had they remained at home. It was our sad duty to pronounce the verdict "dead" upon a poor emaciated consumptive who succumbed in going over the divide. Such cases which increase the local death-rate in health resorts are attributed by the authorities to the ignorance of their attending physicians back east. It may be just in some cases, but of far greater influence in enticing these wrecks of humanity to seek the climatic cure as a last resort are the intemperate and unwarranted claims boldly advertised by interested parties in the west. The climatic cure is legitimate therapeusis under certain conditions. The first essential is an early diagnosis. The second is the ability to support oneself in idleness for a period sufficiently long to insure a complete cure, or until work can be safely undertaken; and the third is a willingness to remain permanently in a region inimicable to the redevelopment of the malady from which one has suffered. Under such conditions and under none others is a physician warranted in separating a sick person from his home and his family.

528 South Salina Street.

